# Intersubtype Reassortments of H5N1 Highly Pathogenic Avian Influenza Viruses Isolated from Quail

**DOI:** 10.1371/journal.pone.0149608

**Published:** 2016-02-22

**Authors:** Tinh Huu Nguyen, Van Thai Than, Hien Dang Thanh, Vu-Khac Hung, Duc Tan Nguyen, Wonyong Kim

**Affiliations:** 1 Department of Microbiology, Chung-Ang University College of Medicine, Seoul, South Korea; 2 Central Vietnam Veterinary Institute, Nha Trang, Vietnam; Zhejiang University, CHINA

## Abstract

H5N1 highly pathogenic avian influenza (HPAI) viruses are considered a threat to national animal industries, causing production losses and high mortality in domestic poultry. In recent years, quail has become a popular terrestrial poultry species raised for production of meat and eggs in Asia. In this study, to better understand the roles of quail in H5N1 viral evolution, two H5N1-positive samples, designated A/quail/Vietnam/CVVI-49/2010 (CVVI-49/2010) and A/quail/Vietnam/CVVI-50/2014 (CVVI-50/2014), were isolated from quail during H5N1 outbreaks in Vietnam, and their whole genome were analyzed. The phylogenetic analysis reveals new evolutionary variation in the worldwide H5N1 viruses. The quail HA genes were clustered into clades 1.1.1 (CVVI-49/2010) and clade 2.3.2.1c (CVVI-50/2014), which may have evolved from viruses circulating from chickens and/or ducks in Cambodia, mainland of China, Taiwan, Indonesia, and South Korea in recent years. Interestingly, the M2 gene of the CVVI-49/2010 strain contained amino acid substitutions at position 26^L-I^ and 31^S-N^ that are related to amantadine-resistance. In particular, the CVVI-50/2014 strain revealed evidence of multiple intersubtype reassortment events between virus clades 2.3.2.1c, 2.3.2.1b, and 2.3.2.1a. Data from this study supports the possible role of quail as an important intermediate host in avian influenza virus evolution. Therefore, additional surveillance is needed to monitor these HPAI viruses both serologically and virologically in quail.

## Introduction

Avian influenza viruses (AIV) belong to the *Orthomyxoviridae* family. The viral genome consists of eight segments of single-stranded negative RNA, encoding at least 10 well-described functional proteins (PB1, PB2, PA, HA, NP, NA, M1, M2, NS1, and NS2), and five recently identified functional proteins (PB1-F2, PB1-N40, PA-X, PA-N155, and PA-N182) [1]. Hemagglutinin (HA) and neuraminidase (NA) are surface antigenic proteins that play a major role in the host humoral immune response against these viruses [2]. Based on the presence of HA and NA antigens, influenza A viruses are divided into H and N subtypes. To date, 18 HA (H1–H18) and 11 NA (N1–N11) subtypes have been identified in aquatic fowls and bats [3]. This suggests the possibility of genetic reassortment containing different combinations of these genes are possible and result in generating new HN subtypes [1,3].

AIVs cause various pathologies in infected birds, ranging from clinically inapparent, to mild illness, to high fatality. Based on the pathogenicity, AIVs are classified into low pathogenic avian influenza (LPAI) and highly pathogenic avian influenza (HPAI) viruses [4]. Of these, only small number of subtypes H5 and H7 are considered HPAI viruses that cause high mortality in wild birds and domestic poultry worldwide [2,4].

An HPAI H5N1 virus was first identified in 1996 in domestic geese in Guangdong province, China [5]. Since then, the virus has spread rapidly to many other countries in Asia, Europe, and Africa, with major economic repercussions due to millions of poultry deaths, including those from culling procedures [6]. The HPAI H5N1 viruses have diversified into nine distinct clades (clades 0–9) and a large number of subclades. In 2008, clades 0, 3, 4, 5, 6, 8, 9, and several subclades from clade 2 were not detected [7]. By 2014, clades 1, subclades 2.1.3, 2.2, 2.2.1, 2.3.2, 2.3.4, and clade 7 had expanded worldwide, despite efforts to control the viruses [8]. Viruses from subclade 2.3.2 have emerged in parts of Asia, including mainland of China, Vietnam, Hong Kong, Japan, Korea, Laos, Bangladesh, Nepal, Mongolia, and the Tyva Republic; and in eastern Europe, particularly in Bulgaria and Romania [7].

Aquatic birds are known to be the natural reservoirs of influenza A viruses. The HA subtype H1–H16, and NA subtypes N1–N9, and most of their combinations have been identified from aquatic birds [9,10]. In addition, quail have been identified as the most important intermediate hosts of AIVs in recent years because they express both SAα-2,3-linked and SAα-2,6-linked receptors for mammalian and avian influenza A viruses on their epithelial cell surfaces [11,12]. *In vivo* experiments have shown that the respiratory tracts of quail can support the replication of a broad range of influenza viruses, including 14 HA subtypes (H1–H14) from aquatic birds, human-like H1N1 virus, and swine H1N1, H1N2, and H3N2 viruses [13]. In nature, quail can be infected with numerous influenza virus subtypes, including AIV subtypes H3 to H7, H9, and H10; human H1N1 virus; and swine H3N2 viruses [11,14]. HPAI H5N1 surveillance studies have shown that quail are infected with HPAI H5N1 viruses in many regions of Asia, including mainland of China, Hong Kong, Indonesia, South Korea, Thailand, and Vietnam, where quail are often found intermingling with other poultry in live bird markets [15–20]. Whole genomic characterization of the three quail HPAI H5N1 clade 4 viruses identified in China indicated that quail may play a role in the evolution of AIVs, because of their ability to be infected by and to transmit H5N1 viruses among poultry, wild birds, and humans [21].

Vietnam is a tropical country that is located on the eastern margin of the Indochinese Peninsula. Agriculture plays a critical role in the national economy, and animal production contributes about 32% to the total agricultural gross domestic product. Since their first detection in 2001, HPAI H5N1 viruses continue to be highly contagious, affecting a large number of wild birds and domestic poultry in this country in recent years [22,23]. HPAI H5N1 viruses in Vietnam have been classified into several genetic groups. Although some H5N1 clades disappear a short time after their emergence, two major clades, clade 1 and clade 2, are still in circulation. Many subclades of clades 1 and 2 have subsequently been identified in poultry [24–27]. Studies have shown that, by 2012, clade 1.1 viruses were dominant among AIVs circulating in southern Vietnam, while viruses isolated from influenza cases in the northern and other regions belonged to clade 2.3.2.1 [28,29].

Quail is a popular terrestrial domestic poultry species raised for meat and eggs in Vietnam. HPAI H5N1 viruses were identified in infected quail in 2004–2005 in the Kien Giang and Tra Vinh provinces in the southern region of Vietnam [30]. However, they have not been fully genetically or phylogenetically characterized. Interestingly, in Vietnam, quails are usually maintained by natural poultry farming practices (e.g., backyard or grazing frame methods) that may contribute to the persistence and spread of influenza viruses throughout the country. In this study, the full genomes of two HPAI H5N1 virus strains isolated from H5N1 outbreaks on quail farms in Khanh Hoa province in 2010 and 2014, a south-central region of Vietnam were characterized in order to determine the genetic relatedness of these strains to reference AIV strains and to describe the possible role of quail in the evolution of AIVs in Vietnam.

## Materials and Methods

### Ethics statement

Data in this study were obtained from case-investigation reports at the Central Vietnam Veterinary Institute (CVVI), Nha Trang, Vietnam and being reported to the authorities. The accurate poultry farm information was supplied by the owners with none of the field studies involve endangered or protected species. Ethics approval for the animal experiments was obtained from the National Institute of Veterinary Research (NIVR) Ethics Committee, Hanoi, Vietnam.

### Sample collection

Two HPAI H5N1 virus strains, designated A/quail/Vietnam/CVVI-49/2010 (CVVI-49/2010) and A/quail/Vietnam/CVVI-50/2014 (CVVI-50/2014), were isolated from H5N1 outbreaks in quail in a passive surveillance program in Khanh Hoa province, a south-central region of Vietnam. These outbreaks occurred on quail farms in January 2010 and March 2014, and killed 476/2300 (20.7%) and 250/900 (27.8%) of the quail in each farm. The clinical signs in infected quail included depression, seizures, ataxia, neurological signs, and green, diarrheal feces. In most cases, two sick or dead quail from each farm were collected and transported to the Central Vietnam Veterinary Institute. Tissue samples from the brain, lung, spleen, bronchus, and intestine were collected and stored at −80°C for further examination. All handling tissue samples and viral cultures were carried in the Biosafety Level 3 laboratory.

### Virus isolation in embryonated chicken eggs

The tissue samples were homogenized in phosphate buffer saline (PBS; pH 7.4) with antibiotics and clarified by centrifugation at 400 × *g* for 10 min. Supernatants were collected and filtered using a 0.45-μm sterile syringe filter (Corning Costar, Corning, NY, USA). Viruses were isolated using embryonated chicken eggs according to the World Health Organization (WHO) manual [31]. Briefly, 100 μl of each sample was inoculated into the amniotic cavity of three 10-day-old specific pathogen-free embryonated chicken eggs, and incubated at 37°C for 48 hrs. Virus was then harvested and stored at −80°C for further examinations.

### RNA extraction and real-time RT-PCR

The pooled tissue samples from each farm were homogenized in phosphate buffer saline (PBS; pH 7.4) and clarified by centrifugation at 400 × *g* for 10 min. Viral RNA was extracted using a QIAamp Viral RNA Mini Kit (Qiagen, Valencia, CA, USA), according to the manufacturer’s instructions. Extracted RNA was resuspended in RNase-free water and stored at −80°C until analysis. Real-time RT-PCR was performed according to the WHO manual [32]. Briefly, the QuantiTech Probe RT-PCR Kit (Qiagen) was used with 12.5 μl of Master Mix, 1.5 μl each of forward and reverse primers (10 μM), 0.5 μl of probe (5 μM), 0.25 μl of QuantiTect^®^RT Mix, 3.75 μl of RNAase free water, and 5.0 μl of RNA template in a 25-μl total volume. Using an ABI 7500 real-time thermocycler (Applied Biosystems, Foster City, CA, USA), reverse transcription was carried out for 30 minutes at 50°C, followed by polymerase activation for 15 minutes at 95°C. Denaturation for 15 seconds at 94°C and annealing-extension for 1 minute at 56°C were performed for 45 cycles to obtain cycle threshold (C*t*) values.

### Reverse transcription-polymerase chain reaction (RT-PCR)

The SuperScript III First-Strand Synthesis Super Mix (Invitrogen, Carlsbad, CA, USA) was used to prepare cDNA from the extracted RNA with Uni12 primer (5′-AGCRAAAGCAGG-3′). The reaction was carried out at 42°C for 60 min, followed by 72°C for 10 min. The full lengths of eight gene segments, HA, NA, PB2, PB1, PA, NP, M, and NS, were amplified as described previously [33,34] (S1 Table). Each PCR product was separated on a 1.2% SeaKem LE agarose gel (FMC, Rockland, ME, USA) and stained with ethidium bromide. The gels were viewed on a Gel Doc XR image analysis system (BioRad, Hercules, CA, USA).

### Nucleotide sequencing and sequence analysis

The amplified PCR products were purified using a QIAquick Gel Extraction Kit (Qiagen). RT-PCR primers were used for direct sequencing of the HA, NA, PB2, PB1, PA, NP, M, and NS genes using a BigDye Terminator Cycle Sequencing Kit and an ABI 3730 DNA sequencer (Applied Biosystems). Walking sequencing primers were designed to obtain the sequences of the 5′ and 3′ ends of each gene (S1 Table). The resulting nucleotide and deduced amino acid sequences were aligned using the Clustal_X 2.1 program [35] and Lasergene software (DNASTAR; Madison, WI, USA), with parameters set based on H5N1 viral sequences in the NCBI GenBank database. The nucleotide sequences obtained in this study were deposited in the GenBank database under the accession numbers KP872889–KP872904.

### Phylogenetic analysis

The nucleotide sequences of the HA, NA, PB2, PB1, PA, NP, M, and NS genes obtained were compared against representative gene sequences from available HPAI H5N1 sequences in the GenBank database. The viral clades were defined follow the manual of the WHO⁄OIE⁄FAO H5N1 Evolution Working Group [7]. Phylogenetic trees were constructed using the neighbor-joining algorithm in the PHYLIP suite and the Kimura two-parameter model using MEGA 6.06 software [36–38]. Evolutionary distances for the neighbor-joining analyses were based on the model described by Jukes and Cantor [39]. Tree topology was evaluated by a bootstrap re-sampling method, with 1000 replicates of the neighbor-joining dataset, using the SEQBOOT and CONSENSE programs in PHYLIP [38].

## Results

### Identification of HPAI H5N1 cases

**Fig 1:**
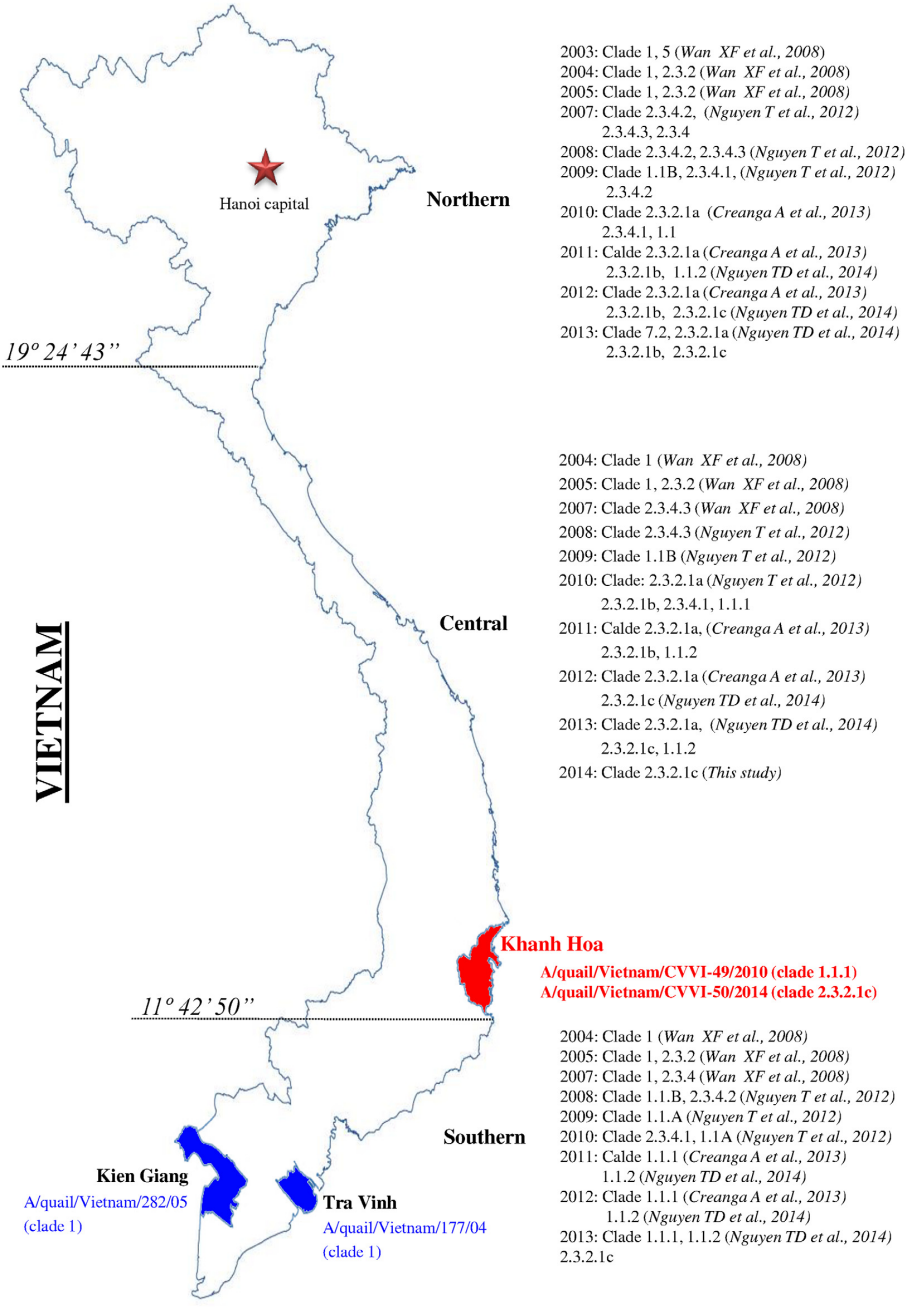
Map of Vietnam showing the study areas. The HPAI H5N1 outbreaks in quail were located in the highlighted provinces (red, green). Text on the right indicates specific HPAI H5N1 outbreaks and the HPAI H5N1 virus types circulating in northern, central, and southern regions of Vietnam between 2003 and 2014.

The quail was dissected at CVVI to observe the clinical and pathological features of AIV infection. Quail with suspected AIV infections all displayed signs of acute phase disease. Internal organs, including the lung, liver, pancreas, and intestines, exhibited extreme swelling and hemorrhage. Viral RNA was extracted from pooled tissues. AIVs were identified by real-time RT-PCR of the M gene, showing C*t* values between 17.6 and 22.6. The HA and NA genes of these two AIVs belonged to the H5 and N1 subtypes, respectively. The viruses were propagated in embryonated chicken eggs. The two H5N1 viruses that were isolated were given the strains designations of A/quail/Vietnam/CVVI-49/2010 (CVVI-49/2010) and A/quail/Vietnam/CVVI-50/2014 (CVVI-50/2014) (Fig 1).

### Phylogenetic analysis

**Fig 2:**
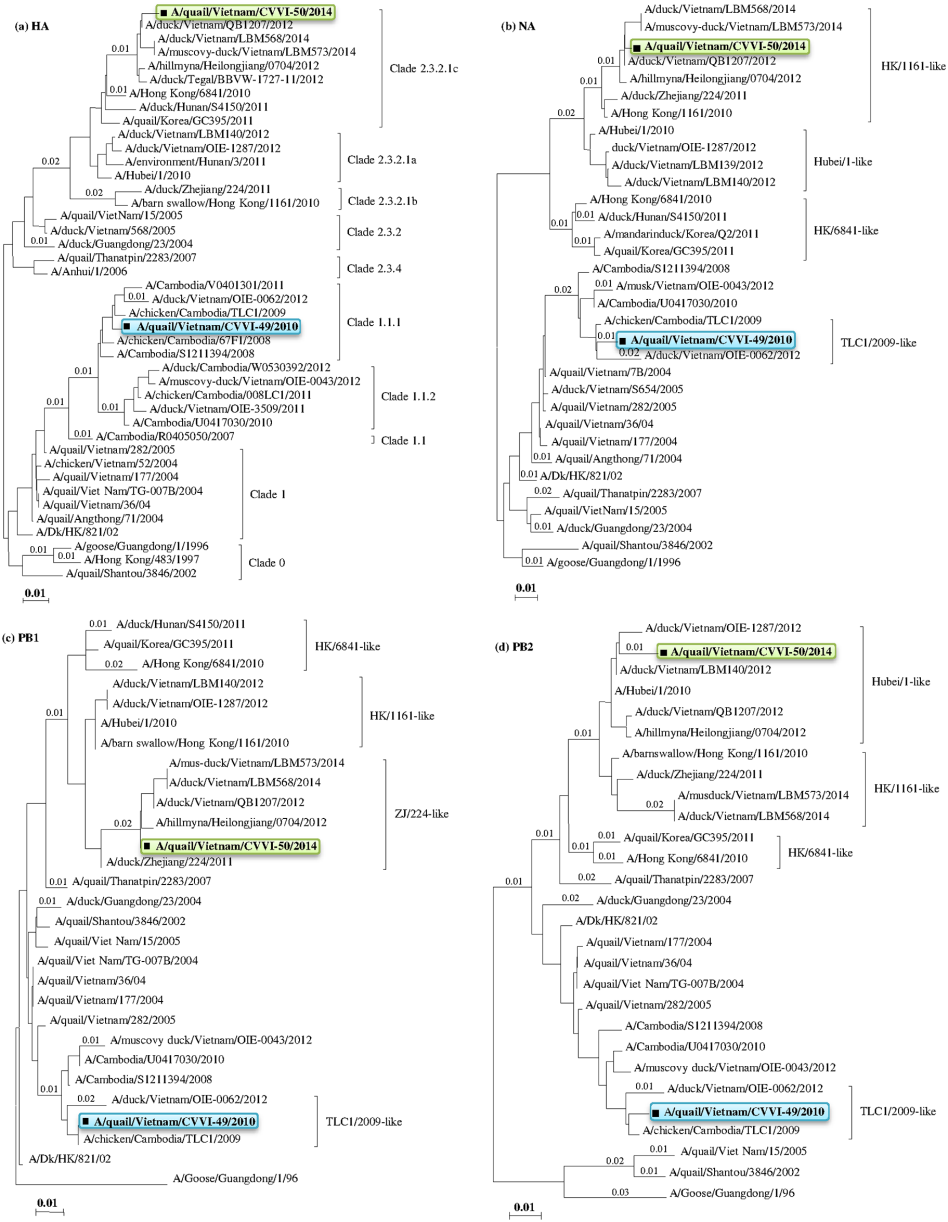
Phylogenetic trees from neighbor-joining analyses of the (a) HA genes, (b) NA genes, (c) PB1 genes, and (d) PB2 genes of the HPAI H5N1 study strains and reference strains available in the GenBank database. The tree topology was constructed using the MEGA 6.06 program and bootstrap values were obtained from 1,000 resampled datasets. HPAI H5N1 virus clade names were determined based on specific criteria and reference strains, as described by the World Health Organization, World Organization for Animal Health, and Food and Agricultural Organization. The study strains are marked in bold and black squares.

Phylogenetic trees for all eights genes, including HA, NA, M, PB2, PB1, PA, NP, and NS, were constructed to examine genetic relatedness using sequences obtained in this study and available sequences in the GenBank database. Phylogenetic analysis indicated that the HA genes of the CVVI-49/2010 and CVVI-50/2014 strains were clustered into clade 1.1.1 (A/chicken/Cambodia/TCL1/2009-like) and clade 2.3.2.1c (A/HongKong/6841/2010-like), respectively (Fig 2a). The CVVI-49/2010 strain was closely related to strains isolated in Cambodia and Vietnam between 2008 and 2012, with a nucleotide (nt) sequence identity to the A/chicken/Cambodia/TCL1/2009 strain of 99.2%. CVVI-50/2014 was closely related to strains isolated in mainland of China, Taiwan, Indonesia, and South Korea, with a nt sequence identity to the A/duck/Vietnam/QB1207/2012 strain of 99.2% (S2 Table).

Phylogenic analysis of the NA gene indicated that the CVVI-49/2010 and CVVI-50/2014 strains fall into two different genogroups, TCL1/2009-like (clade 1.1.1) and HK/1161-like (clade 2.3.2.1b), respectively (Fig 2b). This result may indicate that the NA gene of the CVVI-50/2014 (clade 2.3.2.1c) strain resulted from a reassortment event between clade 2.3.2.1c and 2.3.2.1b viruses (Fig 2a and 2b). Similar to the HA genes, the nt sequences of the NA genes of the CVVI-49/2010 and CVVI-50/2014 strains showed the highest identities to NA genes of the A/chicken/Cambodia/TCL1/2009 and A/duck/Vietnam/QB1207/2012 strains, sharing 99.0% and 99.7% of their nt sequences, respectively (S2 Table).

**Fig 3:**
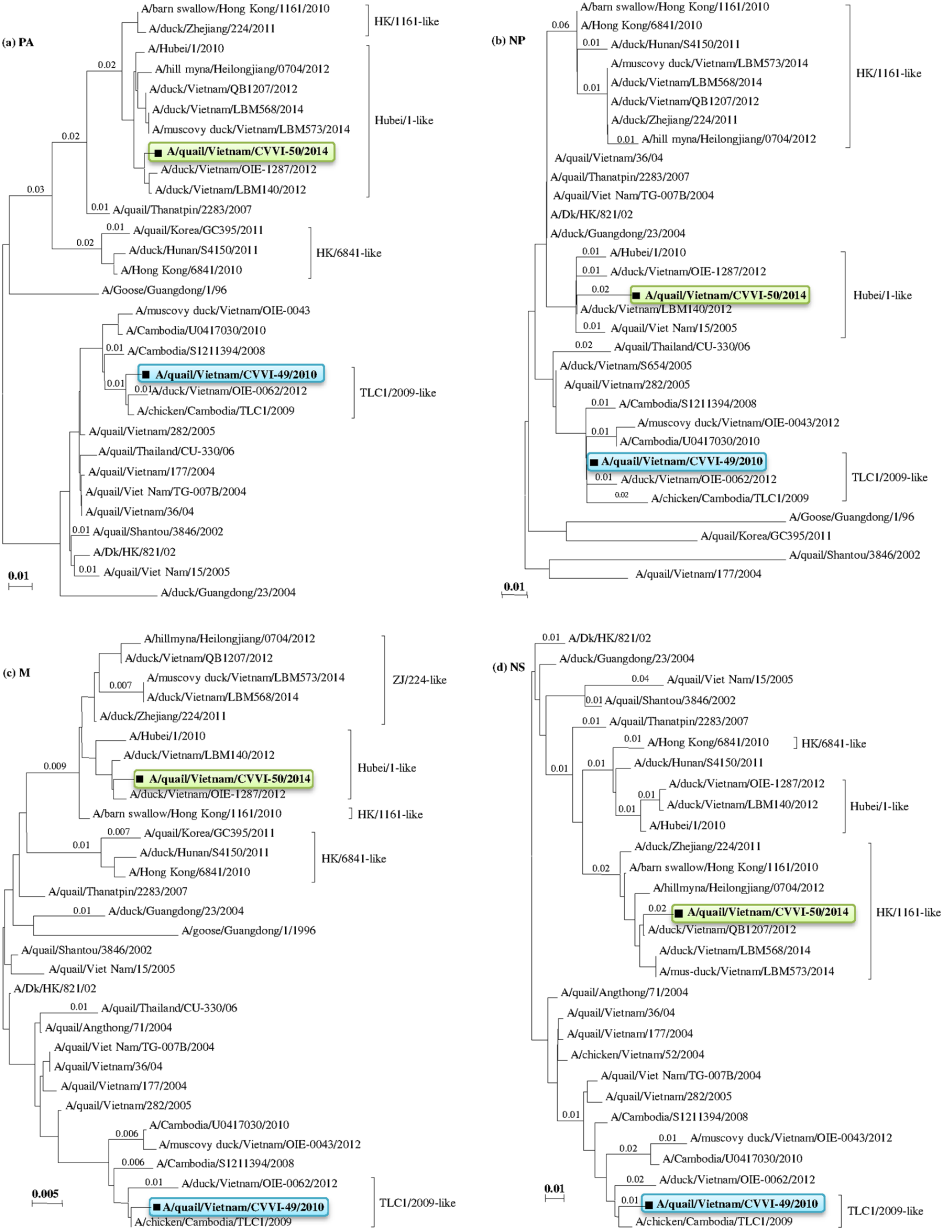
Phylogenetic trees from neighbor-joining analyses of the (a) PA genes, (b) NP genes, (c) M genes, and (d) NS genes of the HPAI H5N1 study strains and reference strains available in the GenBank database. The tree topology was constructed using the MEGA 6.06 program and bootstrap values were obtained from 1,000 resampled datasets. HPAI H5N1 virus clade names were determined based on specific criteria and reference strains, as described by the World Health Organization, World Organization for Animal Health, and Food and Agricultural Organization. The study strains are marked in bold and black squares.

The internal genes of the CVVI-49/2010 strain were most closely related to the A/chicken/Cambodia/TLC1/2009 strain (clade 1.1.1), sharing nt sequence identities of 99.2%, 99.0%, 99.2%, 99.2%, 99.6%, and 98.9% for the PB1, PB2, PA, NP, M, and NS genes, respectively (Figs 2c, 2d and 3a–3d, S2 Table). Interestingly, for the CVVI-50/2014 strain (clade 2.3.2.1c), the majority of the internal genes (PB1, PB2, PA, NP, and M) were found to share a common ancestor with the A/Hubei/1/2010 prototype strain (clade 2.3.2.1a) (Figs 2c, 2d and 3a–3c), while the NS gene was found to share a common ancestor with the A/barn-swallow/Hong Kong/1161/2010 prototype strain (clade 2.3.2.1b) (Fig 3d). These results indicate that the CVVI-50/2014 strain may have resulted from multiple reassortment events between clade 2.3.2.1c, clade 2.3.2.1a, and clade 2.3.2.1b viruses.

### Genetic analysis

Results obtained from molecular analysis of the HA genes are shown in Table 1. The HA cleavage site of the CVVI-49/2010 strain showed the REGRRKKR/G sequence, while the CVVI-50/2014 strain showed the RERRRKR/G sequence, indicating that an amino acid at position 329 was deleted. These results identified the CVVI-49/2010 and CVVI-50/2014 strains as H5N1 HPAI viruses. The receptor biding sites at positions 222 and 224 displayed a glutamine (Q) and glycine (G), respectively, suggesting that these viruses retain their affinities for avian cell surface receptors (SAα-2,3-linked receptor) [40]. Compared to the A/goose/Guangdong/1/1996 strain, the glycosylation sites in the CVVI-49/2010 and CVVI-50/2014 strains contain amino acid substitutions at positions 140^R-K,N^, 154^N-D^, 155^S-N^, and 156^A-T^ (Table 1).

**Table 1:** Deduced amino acid sequences of the CVVI-49/2010 and CVVI-50/2014 strains relative to genetically related strains

Strains	HA					NS					M1		PB2	
	Cleavage site	Glycosylation site		Receptor binding site		Amino acids position				C-terminal	Amino acid position			
	323–332	140–142	154–156	222	224	80–84	42	92	149		30	215	627	701
A/quail/Vietnam/CVVI-49/2010	REGRRKKR/G	KSS	NST	Q	G	Deletion	S	D	A	ESEV	D	A	E	D
A/quail/Vietnam/CVVI-50/2014	RERRRK—R/G	NSS	DNA	Q	G	Deletion	S	E	A	ESEV	D	A	E	D
A/goose/Guangdong/1/1996	RERRRKKR/G	RSS	NSA	Q	G	No deletion	A	D	A	ESEV	D	A	E	D
A/Dk/HK/821/02	IERRRKKR/G	KSS	NSA	Q	G	Deletion	S	D	A	ESEV	D	A	E	D
A/quail/Vietnam/36/2004	RERRRKKR/G	KSS	NST	Q	G	Deletion	S	D	A	ESEV	D	A	E	D
A/quail/Vietnam/177/2004	RERRRKKR/G	KSS	NST	Q	G	Deletion	S	D	A	ESEV	D	A	E	D
A/quail/Vietnam/TG-007B/2004	RERRRKKR/G	KSS	NST	Q	G	Deletion	S	D	A	EPEV	D	A	E	D
A/quail/Vietnam/15/2005	RERRRK—R/G	KSS	NDA	Q	G	Deletion	S	D	A	ESEV	D	A	E	D
A/quail/Vietnam/282/2005	RERRRKKR/G	KSS	NST	Q	G	Deletion	S	D	A	ESEV	D	A	E	D
A/Cambodia/R0405050/2007	REGRRKKR/G	KSS	NNS	Q	G	Deletion	S	D	A	ESEV	D	A	E	D
A/Cambodia/S1211394/2008	REGRRKKR/G	KSS	NST	Q	G	Deletion	S	D	A	ESEV	D	A	E	D
A/Hubei/1/2010	RERRRK—R/G	KSS	DNA	Q	G	Deletion	S	D	A	ESEV	D	A	E	D
A/Hong Kong/6841/2010	RERRRK—R/G	NSS	DNA	Q	G	Deletion	S	D	A	ESEV	D	A	E	D
A/barn swallow/HongKong/1161/2010	IERRRKKR/G	NSS	DNA	Q	G	Deletion	S	E	A	ESEV	D	A	E	D

*HA-Haemagglutinin, NS-Non-structural proteins, M1-Matrix protein M1, and PB2-Polymerase PB2. A-Alanine, D-Aspartic acid, E-Glutamic acid, G-Glycine, I-Isoleucine, K-Lysine, N-Asparagine, Q-Glutamine, R-Arginine, S-Serine, T-Threonine, V-Valine.

Mutations at amino acid positions 42^P-S^, 92^D-E^, and 149^V-A^ of the NS1 protein have been reported to increase the virulence of the H5N1 viruses in different hosts [41–44]. The CVVI-49/2010 and CVVI-50/2014 strains both displayed 42^P-S^ and 149^v-A^ mutations, and, additionally, the CVVI-50/2014 strain contained a 92^D-E^ mutation (Table 1). In addition, five amino acid deletions at positions 80–84, an ESEV sequence at the NS1 C-terminal region, and a PDZ-domain motif were found that could indicate the study strains are highly virulent [44] (Table 1). No genetic marker associated with virulence in the PB2 protein (627^E-K^ and 701^D-N^) in mammals was found in these two strains [45,46].

Compared to the A/goose/Guangdong/1/1996 reference strain, the NA sequences of CVVI-49/2010 and CVVI-50/2014 contain a 20-amino acid deletion at positions 49–68 in the NA stalk region (Table 2). These deletions have been related to AIV virulence and the ability of AIVs to be transmitted from water fowl to terrestrial poultry [47]. No amino acid substitutions were found in the NA region of the study strains. Interestingly, two amino acid mutations, 26^L-I^ and 31^S-N^, were found in the M2 sequences that are known to be related to amantadine resistance [48] (Table 2).

**Table 2:** Antiviral resistance markers in the NA and M2 genes of the CVVI-49/2010 and CVVI-50/2014 strains relative to some genetically related strains.

Strains	NA									M2				
	Stalk region	Amino acids position								Amino acids position				
		116	117	119	150	223	247	275	295	26	27	30	31	34
A/quail/Vietnam/CVVI-49/2010	20 aa deletion	V	I	E	K	I	S	H	N	I	V	A	N	G
A/quail/Vietnam/CVVI-50/2014	20 aa deletion	V	I	E	K	I	S	H	N	L	V	A	S	G
A/goose/Guangdong/1/1996	No deletion	V	I	E	K	I	S	H	N	L	V	A	S	G
A/Dk/HK/821/02	20 aa deletion	V	I	E	K	I	S	H	N	L	V	A	S	G
A/quail/Vietnam/TG-007B/2004	20 aa deletion	V	I	E	K	I	S	H	N	L	V	A	N	G
A/quail/Vietnam/36/2004	20 aa deletion	V	I	E	K	I	S	H	N	L	V	A	N	G
A/quail/Vietnam/177/2004	20 aa deletion	V	I	E	K	I	S	H	N	L	V	A	N	G
A/quail/Vietnam/15/2005	20 aa deletion	V	I	E	K	I	S	H	N	L	V	A	S	G
A/quail/Vietnam/282/2005	20 aa deletion	V	I	E	K	I	S	H	N	L	V	A	N	G
A/Cambodia/R0405050/2007	20 aa deletion	V	I	E	K	I	S	H	N	I	V	A	N	G
A/Cambodia/S1211394/2008	20 aa deletion	V	I	E	K	I	S	H	N	I	V	A	N	G
A/Hubei/1/2010	20 aa deletion	V	I	E	K	I	S	H	N	L	V	A	S	G
A/HongKong/6841/2010	20 aa deletion	V	I	E	K	I	S	H	N	L	V	A	S	G
A/barns wallow/HongKong/1161/2010	20 aa deletion	V	I	E	K	I	S	H	N	L	V	A	S	G

## Discussion

AIVs in land-based domestic poultry and mammalian species, including humans, can evolve rapidly [49]. The variety of viral reservoirs, especially aquatic birds and domestic poultry, may suggest why these viruses persist and are widespread in many countries. HPAI viruses, influenza virus subtypes H5 and H7, have caused high mortality in wild birds and domestic poultry around the world. In Vietnam, HPAI H5N1 viruses were first isolated in 2001; since then, these viruses have become endemic and caused devastating economic repercussions due to millions of poultry deaths, including those from culling [25,26]. The role of quail in the evolution of HPAI H5N1 viruses, and specifically, their ability to maintain and spread these viruses to difference species, was elucidated by several HPAI H5N1 surveillance studies, as well as *in vivo* and *in vitro* examinations [12,13,18,21,50]. Information on HPAI H5N1 outbreaks in quail in Vietnam is limited [30]. This study provides the first characterization of the viral genomes of two HPAI H5N1 viruses isolated from quail H5N1 outbreaks in 2010 and 2014 in Khanh Hoa province located in south-central Vietnam. The data reported here suggests a complex evolutionary picture of HPAI H5N1 viruses that have been circulating Vietnam in recent years, as well as indicate a likely role for quail in the evolution of HPAI viruses in the country.

Phylogenetic analysis of the HA genes indicated that the two study strains, CVVI-49/2010 and CVVI-50/2014, were separated into two different clades, clade 1.1.1 and clade 2.3.2.1c, respectively. Six HA clades have been circulating in Vietnam since 2007, including clades 1.1, 2.3.2.1, 2.3.4.1, 2.3.4.2, 2.3.4.3, and 7.1 [26]. Clade 1 viruses were detected in the northern and central regions of Vietnam between 2003 and 2007, and were subsequently replaced by clade 2.3.4 viruses after 2005 [25]. Clades 1.1.1 and 1.1.2 have emerged as a result of the evolution of clade 1, with clade 1.1.2 viruses persistently circulating in southern Vietnam [51,52]. In 2010, viruses from clade 2.3.2.1 that further evolved into subclades 2.3.2.1 (a,b,c), were identified in the north and have since rapidly spread to the entire country [53]. Detection of clades 1.1.1 and 2.3.2.1c in quail in Khanh Hoa province indicate that quail are susceptible hosts and that they may play important roles in maintaining and spreading HPAI H5N1 viruses among poultry and wild birds, with the potential for human infections [21].

Viral mutation and reassortment are the major evolutionary strategies of influenza viruses in response to immune and environmental pressures, resulting in genetic divergence, the potential for virulence enhancement, and new HPAI outbreaks [54]. Whole genome analyses of AIVs provide an excellent platform for determining the interspecies evolutionary relationships of these viruses [55]. Genetic analysis of HPAI H5N1 viruses circulating in Hong Kong in 1997 (A/Hong Kong/156/97) suggested that this virus strain probably originated from reassortment events between H9N2 (A/quail/HK/G1/97-like), H5N1 (A/goose/Guangdong/1/1996-like), and H6N1 (A/teal/HK/W312/97-like) viruses [56]. In this study, strain CVVI-50/2014 revealed evidence of intersubtype reassortment events between virus clades 2.3.2.1c, 2.3.2.1b, and 2.3.2.1a. The HA gene clustered with clade 2.3.2.1c (A/HongKong/6841/2010-like), while the NA and NS genes clustered with clade 2.3.2.1b (HK/1161-like), while the PB1, PB2, PA, NP, and M genes clustered with clade 2.3.2.1a (A/Hubei/1/2010). In contrast, the CVVI-49/2010 strain shared a common ancestor with the original clade 1.1.1 circulating in Cambodia and Vietnamese. Analysis of the amino acid (aa) sequence revealed that the two study strains shared high identity to the duck and chicken HPAI H5N1 viruses isolated in recently years, rather than with quail HPAI H5N1 viruses, suggesting that the study strains were spread from chickens or ducks to quail. These results support the idea that quail may play an important role in the evolution of HPAI H5N1 viruses in Vietnam [57, 58]. Further analyses will be needed to understand whether or not these strains can be transmitted to other adapted species.

Further analysis revealed that the quail were infected with HPAI H5N1 viruses with common motifs of multiple dibasic amino acids at cleavage sites that have been associated with high pathogenicity and that may be related to the high mortality rates seen at these farms. Quail are terrestrial poultry that should be considered potential intermediate hosts for influenza viruses because of their susceptibility to both mammalian and AIV subtypes [13]. Substitutions at aa positions 42^A-S^ and 92^D-E^ in the NS1 protein that are associated with increased virulence of AIVs in chickens and mice as well as inhibition of host immune responses were found in the study strains [42]. In addition, quails are usually raised in close contact with humans and their population is increasing in Vietnam, posing a concern for public health in areas where HPAI H5N1 viruses are circulating.

Humans infected by HPAI H5N1 viruses are of global public health concern. Worldwide, approximately 667 laboratory-confirmed human cases of H5N1 virus infections were reported during the 2003–2014 transmission season, resulting in 393 deaths [59]. Vietnam is among the countries with the highest rates of human H5N1 infections, with a reported 127 laboratory-confirmed human cases; of these cases, 62 were fatal [60]. Recent human HPAI pandemics have been associated with resistance to anti-influenza drugs, including adamantanes and neuraminidase inhibitors [61]. Resistance to adamantanes, including amantadine and rimantadine, are likely related to specific amino acid substitutions in the M2 protein at positions 26, 27, 30, 31, and 34, and resistance remains high among circulating influenza A viruses [62]. Four types of neuraminidase inhibitors have been developed, including oseltamivir (Tamiflu), zanamivir (Relenza), peramivir (Rapiacta), and laninamivir (Inavir) [63]. Resistance to neuraminidases are likely related to the amino acid substitutions at positions 275 (275^H-Y^) and 295 (295^N-S^) [63,64]. In strains from this study, no amino acid substitutions that are known to be related to oseltamivir resistance were found. However, the M2 gene of the CVVI-49/2010 strain contained amino acid substitutions at positions 26^L-I^ and 31^S-N^, which may cause amantadine resistance [48]. To maintain treatment options, continued antiviral susceptibility monitoring in H5N1 viruses is needed [48].

In conclusion, this study provided the full genomic characterization of HPAI H5N1 subtype viruses in quail in Vietnam. The results indicate the possibility of multiple genetic reassortment events among HPAI H5N1 virus clades 2.3.2.1c, 2.3.2.1b, and 2.3.2.1a. In addition, markers of resistance to anti-influenza drugs, the adamantanes, were identified. These data provide support for the role of quail as an important intermediate host in AIV evolution. Therefore, additional surveillance is needed to monitor AIVs, serologically and virologically, in quail in Vietnam.

## Supporting Information

S1 Table Primers used for amplifying and sequencing of the eight gene segments in this study. (DOC)

S2 Table Nucleotide sequence identity of the eight gene segments of the CVVI-49/2010 and CVVI-50/2014 strains relative to some genetically related strains. (DOC)
