# Supplementary material for: Intersubtype Reassortments of H5N1 Highly Pathogenic Avian Influenza Viruses Isolated from Quail
Source: PLoS One. 2016 Feb 22;11(2):e0149608. doi: 10.1371/journal.pone.0149608 (PMC4765837; doi:10.1371/journal.pone.0149608)
Supplement: S1 Table — (DOC) [file pone.0149608.s001.doc]

**S1 Table**. Primers used for amplifying and sequencing of the eight gene segments in this study

| Genes | Primer name | Primer sequences (5’-3’) | References |
| --- | --- | --- | --- |
| HA | Bm-HA-1 | TATTCGTCTCAGGGAGCAAAAGCAGGGG | Hoffman *et al*, 2001 |
|  | Bm-NS-890R | ATATCGTCTCGTATTAGTAGAAACAAGGGTGTTTT |  |
|  | H5-699R | CTYTGRTTYAGTGTTGATGT | Lee *et al*, 2001 |
|  | H5-317F | CCAATCCAGCCAATGACCTC | This study |
|  | H5-918F | CCARTRGGKGCKATAAAYTC | Tsukamoto *et al*, 2012 |
|  | H5-1178R | GTCTGCAGC RTAYCCACTYC |  |
| NA | Bm-NA-1 | TATTGGTCTCAGGGAGCAAAAGCAGGAGT | Hoffman *et al*, 2001 |
|  | Bm-NA-1413R | ATATGGTCTCGTATTAGTAGAAACAAGGAGTTTTTT |  |
|  | N1-660F | GACACTATCAAGAGTTGGAGG | This study |
|  | N1-961R | GAGCCATGCCAATTATCCCTG |  |
| M | Bm-M-1 | TATTCGTCTCAGGGAGCAAAAGCAGGTAG | Hoffman *et al*, 2001 |
|  | Bm-M-1027R | ATATCGTCTCGTATTAGTAGAAACAAGGTAGTTTTT |  |
|  | M-370F | CGCACTCAGTTACTCAACCG | This study |
|  | M-771R | TGCATCTGCACTCCCATTCG |  |
| NP | Bm-NP-1 | TATTCGTCTCAGGGAGCAAAAGCAGGGTA | Hoffman *et al*, 2001 |
|  | Bm-NP-1564R | ATATCGTCTCGTATTAGTAGAAACAAGGGTATTTTT |  |
|  | NP-759F | GATGGATCAAGTGCGAGAGAGC | This study |
|  | NP-908R | CCACTGGCCACTGCAAGTCCG |  |
| PB2 | Bm-PB2-1 | TATTGGTCTCAGGGAGCGAAAGCAGGTC | Hoffman *et al*, 2001 |
|  | Bm-PB2-2341R | ATATGGTCTCGTATTAGTAGAAACAAGGTCGTTT |  |
|  | PB2-1250R | TCYTCYTGTGARAAYACCAT | Li *et al*, 2007 |
|  | PB2-1105F | TAYGARGARTTCACAATGGT |  |
| PB1 | Bm-PB1-1 | TATTCGTCTCAGGGAGCGAAAGCAGGCA | Hoffman *et al*, 2001 |
|  | Bm-PB1-2341R | ATATCGTCTCGTATTAGTAGAAACAAGGCATTT |  |
|  | PB1-1262R | TTRAACATGCCCATCATCAT | Li *et al*, 2007 |
|  | PB1-1124F | ARATACCNGCAGARATGCT |  |
| PA | Bm-PA-1 | TATTCGTCTCAGGGAGCGAAAGCAGGTAC | Hoffman *et al*, 2001 |
|  | Bm-PA-2233R | ATATCGTCTCGTATTAGTAGAAACAAGGTACTT |  |
|  | PA-1498R | TNGTYCTRCAYTTGCTTATCAT | Li *et al*, 2007 |
|  | PA-747F | CATTGAGGGCAAGCTTTC |  |
| NS | Bm-NS-1 | TATTCGTCTCAGGGAGCAAAAGCAGGGTG | Hoffman *et al*, 2001 |
|  | Bm-NS-890R | ATATCGTCTCGTATTAGTAGAAACAAGGGTGTTTT |  |
