# Supplementary material for: Intersubtype Reassortments of H5N1 Highly Pathogenic Avian Influenza Viruses Isolated from Quail
Source: PLoS One. 2016 Feb 22;11(2):e0149608. doi: 10.1371/journal.pone.0149608 (PMC4765837; doi:10.1371/journal.pone.0149608)
Supplement: S2 Table — (DOC) [file pone.0149608.s002.doc]

**S2 Table**. Nucleotide sequence identity of the eight gene segments of the CVVI-49/2010 and CVVI-50/2014 strains relative to some genetically related strains

| Genes | A/quail/Vietnam/CVVI-49/2010 | Nt sequence  identity (%) | A/quail/Vietnam/CVVI-50/2014 | Nt sequence  identity (%) |
| --- | --- | --- | --- | --- |
| PB2 | A/chicken/Cambodia/TLC1/2009 *(clade 1.1.1)* | 99.0 | A/duck/Vietnam/LBM140/2012 *(clade 2.3.2.1a)* | 98.5 |
| PB1 | A/chicken/Cambodia/TLC1/2009 *(clade 1.1.1)* | 99.2 | A/duck/Vietnam/QB1207/2012 *(clade 2.3.2.1c)* | 99.0 |
| PA | A/chicken/Cambodia/TLC1/2009 *(clade 1.1.1)* | 99.2 | A/duck/Vietnam/LBM140/2012 *(clade 2.3.2.1a)* | 99.0 |
| HA | A/chicken/Cambodia/TLC1/2009 *(clade 1.1.1)* | 99.2 | A/duck/Vietnam/QB1207/2012 *(clade 2.3.2.1c)* | 99.2 |
| NP | A/chicken/Cambodia/TLC1/2009 *(clade 1.1.1)* | 99.2 | A/duck/Vietnam/LBM140/2012 *(clade 2.3.2.1a)* | 98.9 |
| NA | A/chicken/Cambodia/TLC1/2009 *(clade 1.1.1)* | 99.0 | A/duck/Vietnam/QB1207/2012 *(clade 2.3.2.1c)* | 99.7 |
| M | A/chicken/Cambodia/TLC1/2009 *(clade 1.1.1)* | 99.6 | A/duck/Vietnam/LBM140/2012 *(clade 2.3.2.1a)* | 99.3 |
| NS | A/chicken/Cambodia/TLC1/2009 *(clade 1.1.1)* | 98.9 | A/duck/Vietnam/QB1207/2012 *(clade 2.3.2.1c)* | 98.4 |
